# Stacking resistance to crown gall and nematodes in walnut rootstocks

**DOI:** 10.1186/1471-2164-14-668

**Published:** 2013-10-01

**Authors:** Sriema L Walawage, Monica T Britton, Charles A Leslie, Sandra L Uratsu, YingYue Li, Abhaya M Dandekar

**Affiliations:** 1 Department of Plant Sciences, University of California Davis, One Shields Avenue, Davis, CA, 95616, USA; 2 College of Biological Sciences and Technology, Beijing Forestry University, Beijing, 100083, PR China

**Keywords:** Walnuts, Resistance, Gene stacking, Crown gall, Root lesion nematodes, *Pratylenchus vulnus*, RNAi, Co-transformation

## Abstract

**Background:**

Crown gall (CG) (*Agrobacterium tumefaciens*) and the root lesion nematodes (RLNs) (*Pratylenchus vulnus*) are major challenges faced by the California walnut industry, reducing productivity and increasing the cost of establishing and maintaining orchards. Current nematode control strategies include nematicides, crop rotation, and tolerant cultivars, but these methods have limits. Developing genetic resistance through novel approaches like RNA interference (RNAi) can address these problems. RNAi-mediated silencing of CG disease in walnut (*Juglans regia* L.) has been achieved previously. We sought to place both CG and nematode resistance into a single walnut rootstock genotype using co-transformation to stack the resistance genes. *A*. *tumefaciens*, carrying self-complimentary iaaM and ipt transgenes, and *Agrobacterium rhizogenes*, carrying a self-complimentary Pv010 gene from *P*. *vulnus*, were used as co-transformation vectors. RolABC genes were introduced by the resident T-DNA in the *A*. *rhizogenes* Ri-plasmid used as a vector for plant transformation. Pv010 and Pv194 (transgenic control) genes were also transferred separately using *A*. *tumefaciens*. To test for resistance, transformed walnut roots were challenged with *P*. *vulnus* and microshoots were challenged with a virulent strain of *A*. *tumefaciens*.

**Results:**

Combining the two bacterial strains at a 1:1 rather than 1:3 ratio increased the co-transformation efficiency. Although complete immunity to nematode infection was not observed, transgenic lines yielded up to 79% fewer nematodes per root following *in vitro* co-culture than untransformed controls. Transgenic line 33-3-1 exhibited complete crown gall control and 32% fewer nematodes. The transgenic plants had thicker, longer roots than untransformed controls possibly due to insertion of rolABC genes. When the Pv010 gene was present in roots with or without rolABC genes there was partial or complete control of RLNs. Transformation using only one vector showed 100% control in some lines.

**Conclusions:**

CG and nematode resistance gene stacking controlled CG and RLNs simultaneously in walnuts. Silencing genes encoding iaaM, ipt, and Pv010 decrease CG formation and RLNs populations in walnut. Beneficial plant genotype and phenotype changes are caused by co-transformation using *A*. *tumefaciens* and *A*. *rhizogenes* strains. Viable resistance against root lesion nematodes in walnut plants may be accomplished in the future using this gene stacking technology.

## Background

Crown gall (caused by *Agrobacterium tumefaciens*) and root lesion nematode (*Pratylenchus vulnus*) infestations present a serious challenge to the walnut industry. These problems have resulted in unsalable nursery stock, lower productivity, and increased susceptibility of infected plants to biotic and abiotic stresses. Root lesion nematode is a migratory endoparasite that feeds on the roots of many perennial plants, including fruit and nut trees. In California, it is an important pest of walnut, as all existing walnut rootstocks are susceptible or have incomplete resistance [1,2]. Current nematode control strategies include nematicides, crop rotation, and tolerant cultivars, but each has serious limitations. Crown gall disease is caused by the soil bacterium *A*. *tumefaciens*. At present, crown gall disease is managed using surgical removal of the gall and infected tissues or by complete excavation of the diseased tree [3]. This is costly and time-consuming, especially when many trees are infected. As in many crops, either natural crown gall and *P*. *vulnus* resistance is unavailable to walnut breeders or progress by this approach is slow. Thus, stacking resistance genes using genetic technology is an attractive option to more quickly gain control of this disease and pest problem in walnuts.

RNA interference (RNAi) is a functional genomics tool that has been used successfully for plant-parasitic nematode control [4-6]. RNAi is a process in which double-stranded RNA (dsRNA) triggers the silencing of specific target genes through mRNA degradation. RNAi has been tested in many organisms including mammals, insects, fungi, and plants [7-11]. RNAi-induced suppression of many genes is essential for nematode development, reproduction, or parasitism. In *C*. *elegans*, RNAi can be induced by exogenous dsRNA (100–500 bp long) introduced via soaking, microinjection, or by feeding of dsRNA [12-15]. This RNAi technology, and *C*. *elegans* genome sequencing, now provides new opportunities for new research on plant parasitic nematodes.

RNAi has successfully controlled plant parasitic nematodes such as cyst nematodes [16-19], root-knot nematodes [4,20,21], and recently root lesion nematodes [22]. Delivery of dsRNA to nematode juveniles via soaking has been used successfully to investigate the functions of some genes in cyst nematodes (*Globodera pallida* and *Heterodera glycines*), root-knot nematodes (*Meloidogyne incognita*, *Meloidogyne hapla*, and *Meloidogyne javanica*), and migratory nematodes (*Radopholus similis* and *Bursaphelenchus xylophilus*) [18,21,23-27]. Recently, this technique was used to control RLNs *P*. *thornei* and *P*. *zeae*[22]. There is evidence that plant-delivered siRNA/dsRNA reduces nematode establishment and development [19,21,28,29]. However, there are very few published works to show whether RLNs like *P*. *vulnus* are also controlled using RNAi.

We used self-complimentary constructs based on iaaM and ipt genes from *A*. *tumefaciens* and the Pv010 [GenBank: CV200529] gene from *P*. *vulnus* and an *Agrobacterium rhizogenes* vector to stack RNAi mediated resistance to both crown gall and nematode into walnut. Control of crown gall disease alone using RNAi was successfully achieved previously [30,31]. Pv010 was targeted based on its similarity to a *C*. *elegans* gene whose expression reduces fecundity. Pv010 is orthologous to the *C*. *elegans* prp-8 gene, a spliceosome subunit whose RNAi phenotype includes a sterility or juvenile lethality in *C*. *elegans*[32,33]. Pv194 [GenBank: CV199427] was used as a negative control, since its *C*. *elegans* orthologs have wild-type RNAi phenotypes. Based on the *C*. *elegans* ortholog ttr-51 RNAi phenotype (wild-type), it was predicted that Pv194 RNAi should not reduce nematode populations. *Agrobacterium*-mediated co-transformation was used to insert the crown gall RNAi [30,31] and *P*. *vulnus* RNAi [34] constructs into walnut somatic embryos. Transformed somatic embryos were germinated and roots were challenged with *P*. *vulnus*. Nematode multiplication was examined 60 days after infection. Transformed microshoots were challenged with virulent *A*. *tumefaciens* strain 20W-5A [30]. These genetically modified nematode- and crown gall-resistant walnut plants could help reduce the demand for environmentally harmful nematicides.

## Results

### Creation and selection of somatic embryo lines expressing transgenes

*Agrobacterium* inoculated embryos that remained alive during seven weeks of selection on kanamycin-containing media were tested further to confirm transformation. Their transgenic nature was confirmed using GUS assay and PCR. Some J1 and RR4 co-transformed lines were positive for both nptII and Pv010. The 1:1 CG:Pv010 ratio produced a higher co-transformation efficiency than the 1:3 ratio. When only one bacterial strain was used for transformation, 72% of the embryos were positive for the Pv010 gene and 92% were positive for the Pv194 gene (Table 1). J1 and RR4 transgenic lines and their genotypes determined by PCR are listed in Table 2.

**Table 1:** Molecular analysis of transgenic embryos using Pv010 and nptII primers

**Walnut genotypes and vector combinations**	**Samples PCR tested using nptII and Pv010 primers (no.)**	**Percentage co-transformed or single-vector transformed**
J1 - 1:3 CG:Pv010	77	5/77 (6.49%)^1^
J1 - 1:1 CG:Pv010	23	4/23 (17.39%)^1^
RR4 - 1:3 CG:Pv010	1	1/1 (100%)^1,2^
RR4 - 1:1 CG:Pv010	7	5/7 (71.42%)^1^
J1- Pv 010	25	18/25 (72%)^3^
J1- Pv 194	25	23/25 (92%)^4^

^1^Co-transformed percentage.

^2^The only transgenic line that survived was positive for both Pv10 and nptII.

^3^Single-vector transformed percentage. These samples only tested with Pv010 primers.

^4^Single-vector transformed percentage. These samples only tested with Pv194 primers.

**Table 2:** Genotypes of transformed J1 and RR4 walnut rootstocks determined by PCR

**Walnut genotypes**	**Vector combinations**	**Embryo ID no.**	**GUS**	**nptII**	**Pv010**	**Pv194**	**iaaM/ipt**	**rol A**	**rol B**	**rol C**
J1	1:3	32-2-2	**+**	**+**	**+**	**_**	**+**	**+**	**+**	**+**
		33-3-1	**+**	**+**	**+**	**_**	**+**	**+**	**+**	**+**
		66-1-2	**+**	**+**	**+**	**_**	**+**	**+**	**+**	**+**
		68-3-1	**+**	**+**	**+**	**_**	**+**	**+**	**+**	**+**
		73-2-1	**+**	**+**	**+**	**_**	**+**	**+**	**+**	**+**
	1:1	2-2-1	**+**	**+**	**+**	**_**	**+**	**+**	**+**	**+**
		6-2-1	**+**	**+**	**+**	**_**	**+**	**+**	**+**	**+**
		21-2-1	**+**	**+**	**+**	**_**	**+**	**+**	**+**	**+**
		23-3-2	**+**	**+**	**+**	**_**	**+**	**+**	**+**	**+**
RR4	1:3	38-1-1	**+**	**+**	**+**	**_**	**+**	**+**	**+**	**+**
	1:1	7 -2-1	**+**	**+**	**+**	**_**	**+**	**+**	**+**	**+**
		8 -1-1	**+**	**+**	**+**	**_**	**+**	**+**	**+**	**+**
		23-1-1	**+**	**+**	**+**	**_**	**+**	**+**	**+**	**+**
		44-1-1	**+**	**+**	**+**	**_**	**+**	**+**	**+**	**+**
		73-1-2	**+**	**+**	**+**	**_**	**+**	**+**	**+**	**+**
J1^1^	1^1^	2-1-1	**+**	**+**	**+**	**_**	**_**	**_**	**_**	**_**
		15-3-1	**+**	**+**	**+**	**_**	**_**	**_**	**_**	**_**
		24-2-2	**+**	**+**	**+**	**_**	**_**	**_**	**_**	**_**
		29-3-1	**+**	**+**	**+**	**_**	**_**	**_**	**_**	**_**
		30-2-2	**+**	**+**	**+**	**_**	**_**	**_**	**_**	**_**
	1^1^	8-1-1	**+**	**+**	**_**	**+**	**_**	**_**	**_**	**_**
		13-3-2	**+**	**+**	**_**	**+**	**_**	**_**	**_**	**_**
		15-3-1	**+**	**+**	**_**	**+**	**_**	**_**	**_**	**_**
		24-2-1	**+**	**+**	**_**	**+**	**_**	**_**	**_**	**_**

^**1**^One vector (*A*. *tumefaciens* carrying Pv010 gene or Pv194) used for transformation.

### Transgenic plant challenge with root lesion nematodes

**Figure 1:**
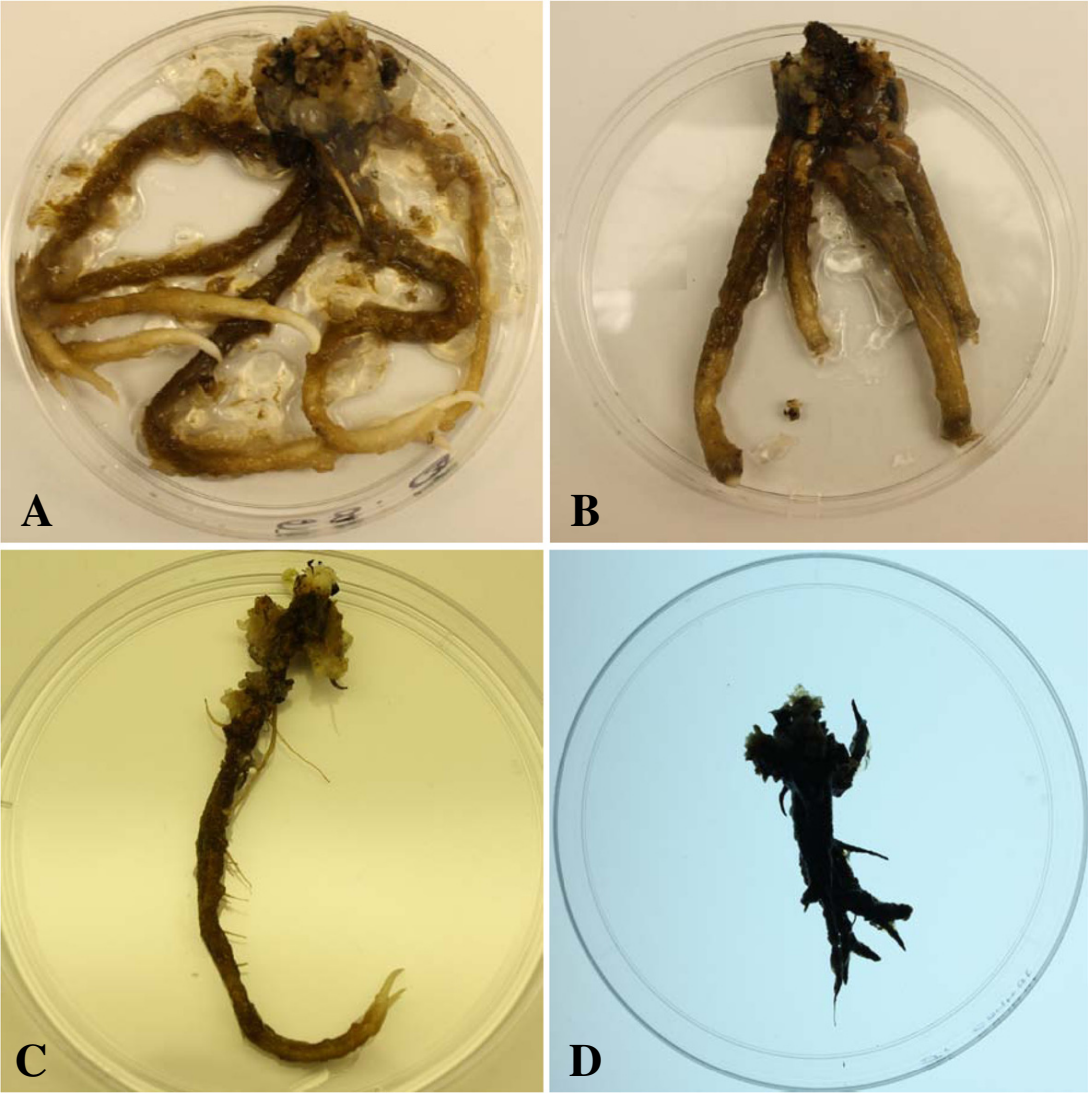
**Roots of J1 somatic embryos transformed with the Pv010 gene. (A)** nematode-resistant roots of line 68-3-1. Roots showed no visible symptoms of nematode damage. **(B)** nematode-damaged roots of line 66-1-2. Roots were damaged by nematodes after two months of infection. **(C)** Single-vector transformed line 29-3-1 with no visible damage. **(D)** Untransformed J1 roots with nematode damage.

**Figure 2:**
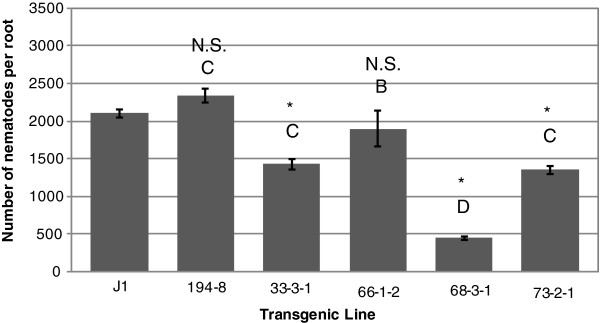
**Inhibition of root lesion nematodes in roots of co**-**transformed somatic embryos of genotype J1.** Nematode infestation is expressed as the number of nematodes recovered from cultures initiated using 100 nematodes per rooted embryo. Nematodes were recovered per root for each transgenic line after two months of *in vitro* co-culture in the dark. Bars represent mean of three replicates (Error bars=S.D). Significant differences from controls are denoted with *. Two lines are significantly different whenever they have no letters in common. Comparisons are considered significant whenever *p*<0.05.

**Figure 3:**
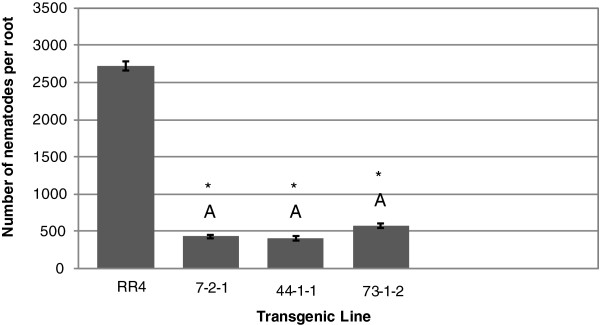
**Inhibition of root lesion nematode in roots of co-transformed somatic embryos of genotype RR4.** Nematode infestation is expressed as the number of nematodes recovered from cultures initiated using 100 nematodes per rooted embryo. Nematodes were recovered per root for each transgenic line after two months of *in vitro* co-culture in the dark. Bars represent mean of three replicates (Error bars=S.D). Significant differences from controls are denoted with *. Two lines are significantly different whenever they have no letters in common. Comparisons are considered significant whenever *p*<0.05.

**Figure 4:**
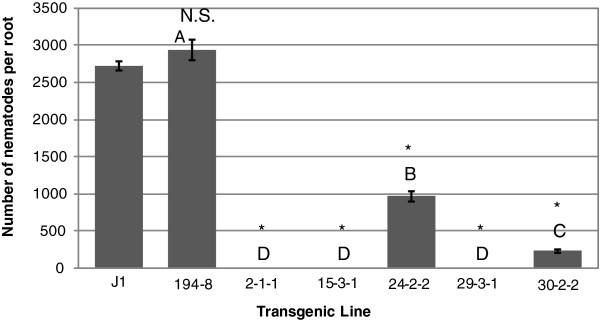
**Inhibition of root lesion nematode in roots of single-vector transformed somatic embryos of genotype J1B.** Nematode infestation is expressed as the number of nematodes recovered from cultures initiated using 100 nematodes per rooted embryo. Nematodes were recovered per root for each transgenic line after two months of *in vitro* co-culture in the dark. Bars represent mean of three replicates (Error bars=S.D). Significant differences from controls are denoted with *. Two lines are significantly different whenever they have no letters in common. Comparisons are considered significant whenever *p*<0.05.

A rapid screening method was used to test for nematode resistance in both co-transformed and single-vector transformed transgenic lines. The nematode population supported by each transgenic line was compared to untransformed (J1 and RR4) and transgenic (Pv194-8) controls. Results of these trials are shown in the figures below (Figures 1, 2, 3 and 4).

The two controls (untransformed J1 and J1 transformed with Pv194-8) and line 66-1-2 were not significantly different in number of nematodes per root. Transgenic line 68-3-1 supported up to 79% fewer nematodes per root following *in vitro* co-culture than untransformed controls. *Invitro* grown roots of this line showed no visible damage due to nematode feeding after two months (Figure 1A). Lines 33-3-1 and 73-2-1 supported ~32% and ~36% fewer nematodes than the control nematode population, respectively. The least effective line, 66-1-2, still had ~10% fewer nematodes than the control nematode population (Figure 2). Roots tips of line 66 showed damage from nematode feeding and had less growth (Figure 1B). RR4 transgenic lines had 79-84% fewer nematodes per root than the untransformed RR4 control (Figure 3). Out of the five single-vector transformed lines tested, three lines (29-3-1, 2-1-1, and 15-3-1) controlled the nematode population completely (100% nematode reduction). Two other lines had ~64% (24-2-2) and ~91% (30-2-2) fewer nematodes relative to the untransformed control (Figure 4) and no visible damage was observed in these roots (Figure 1C) while untransformed roots had damaged, undeveloped, and blackened roots (Figure 1D).

These results show that independent of the rolABC genes inserted into the co-transformed lines, the Pv010 gene effectively controlled nematodes up to 79% in co-transformed lines and 100% in single-vector transformed lines. RolABC genes were introduced into the embryos by the resident T-DNA in the *A*. *rhizogenes* Ri-plasmid used as a vector for plant transformation. In the single-vector transformed lines, there were no rolABC genes inserted with the plasmid as *A*. *tumefaciens* does not carry them.

### Crown gall challenge bioassay

**Figure 5:**
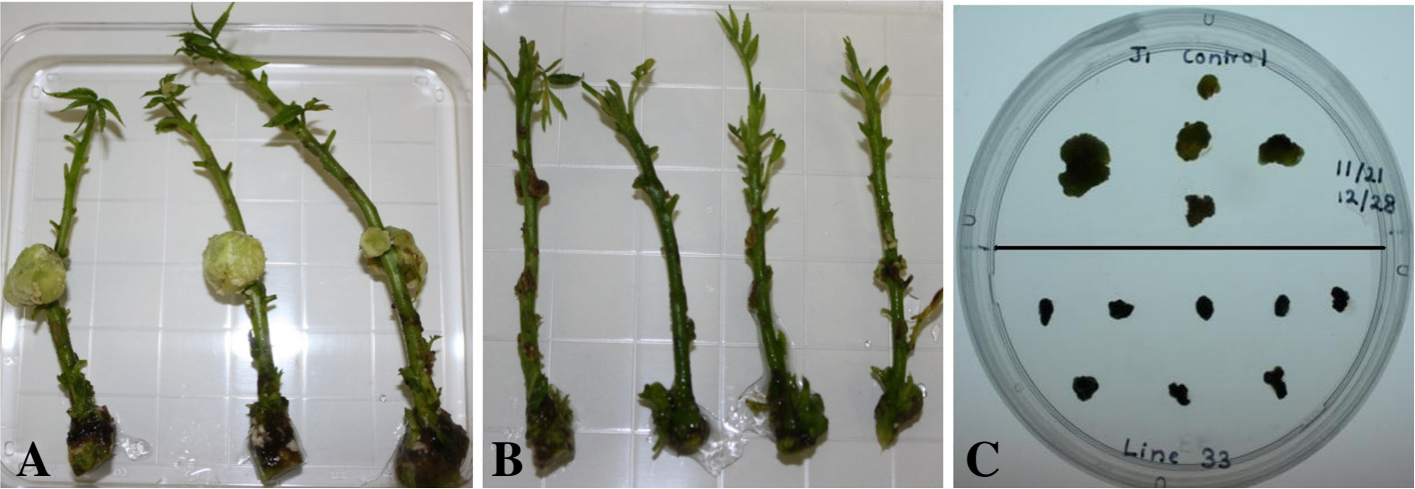
**Suppression of *A*. *tumefaciens *tumorigenesis in oncogene-silenced walnut microshoots.** Walnut microshoots were inoculated with virulent *A*. *tumefaciens* strain 20W-5A. **A)** Inoculation sites of untransformed microshoots formed large, undifferentiated tumors five weeks post-inoculation. **B)** Oncogene-silenced line 33-3-1 exhibited no tumor development. **C)** Small fragments of callus from *A*. *tumefaciens* inoculation sites cultured on hormone-free plant growth medium for five weeks: untransformed callus (upper) and callus derived from transgenic line 33-3-1 (lower).

Oncogene (iaaM and ipt) silencing was screened phenotypically following *in vitro* application of *A*. *tumefaciens* to sixteen microshoots generated from transformed line 33-3-1 or untransformed controls. The amount of undifferentiated callus forming on these tissues after inoculation with virulent *A*. *tumefaciens* strain 20W-5A was assayed five weeks post-inoculation (Figure 5A and B). Untransformed controls formed galls on 14 of 16 microshoots inoculated whereas no galls were formed on shoots of the transformed line 33. Control lines developed green callus at inoculation sites after five weeks. To confirm that this callus was crown gall tissue, small pieces were cultured further on hormone-free DKW medium. While most walnut tissues (including wound callus) will not proliferate on this medium, crown gall tissue is capable of rapid hormone-independent growth. Callus excised from controls displayed vigorous hormone-independent expansion while callus excised from line 33 displayed only minimal growth (Figure 5C). These results indicate crown gall initiation and proliferation is suppressed in transgenic line 33.

### Plant genotype and morphological changes

**Figure 6:**
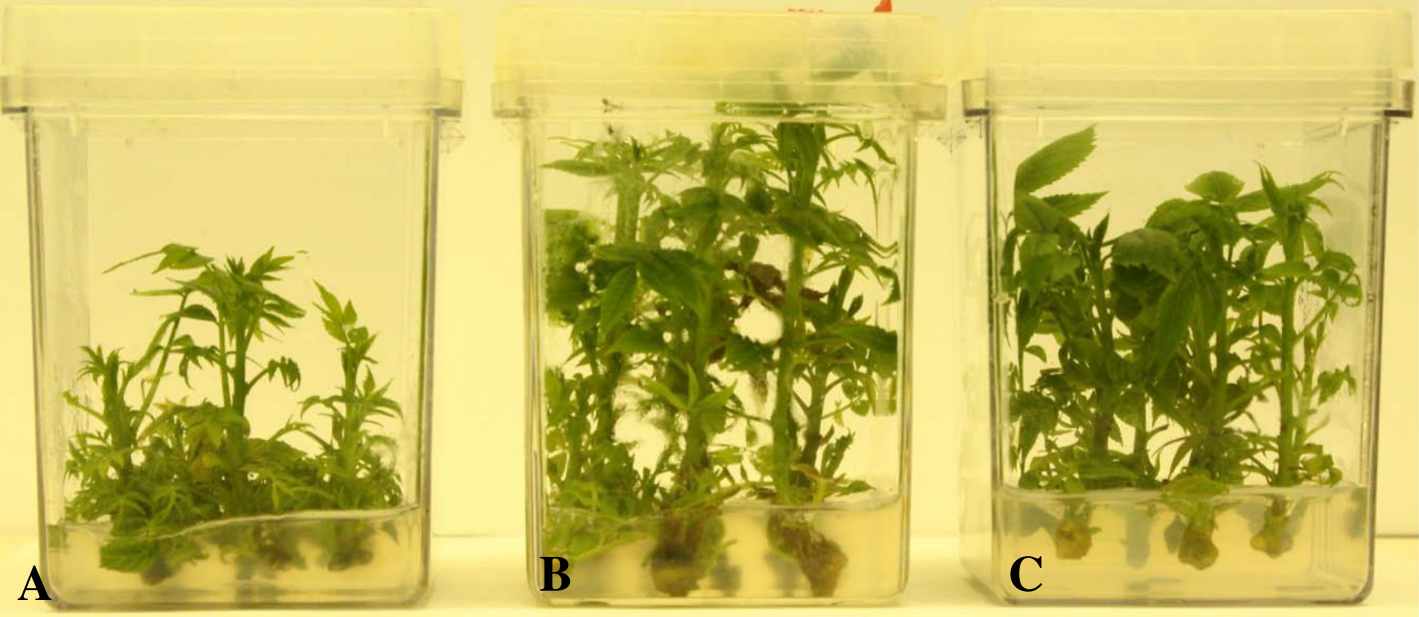
**Morphology of transformed walnut shoots. A)***In vitro*-grown plants of co-transformed 33-3-1. **B)** Untransformed control. Co-transformed plants had stunted growth, shorter internode lengths, and darker green, smaller, and more wrinkled leaves than the control. **C)***In vitro*-grown plants of single-vector transformed line 29-3-1 were phenotypically similar to the untransformed control.

Co-transformation resulted in 15 stable transgenic lines stacked with GUS, nptII, Pv010, iaaM and ipt, and rolABC (rolA+rolB+rolC) in walnut rootstock backgrounds J1 and RR4. The plants transformed with only Pv010 and Pv194 genes did not obtain rol genes but GUS, nptII, Pv010 or Pv194 in rootstock backgrounds J1 (Table 2). To see whether co-transformation changed the phenotype of transformants, the morphology of *in vitro*-grown microshoots of transgenic lines was compared to untransformed microshoots. Shoot height, internode length, leaf size, and leaf appearance were assessed. Major morphological differences were observed in shoots generated using the *A*. *rhizogenes* vector, including stunted growth, shorter internode length, and dark green, small, wrinkled leaves. This was especially apparent in line 33-3-1 (Figure 6A). Similarly, root growth patterns and root length of transgenic and untransformed *in vitro*-grown plantlets were compared. All co-transformed roots had 2–2.5 times greater root diameters than untransformed lines. Shoots and roots obtained from single-vector transformation were similar in phenotype to untransformed plants (Figures 6B and C, Figure 1C and D).

## Discussion

“Gene stacking” was used to generate transgenic walnut lines resistant to both nematodes and crown gall infection. Crown gall resistance has been achieved previously in walnut using the self-complementary pDE00.0201 expression cassette and somatic embryos as explants [30,31]. We used the same vector in co-transformations to produce transgenic line 33-3-1, which exhibited both complete suppression of crown gall and 32% fewer nematodes than untransformed control. Co-transformations were designed to suppress expression of both the oncogenes iaaM and ipt and the *P*. *vulnus* gene Pv010 through RNA interference. Stacking crown gall RNAi genes in combination with a nematode RNAi gene proved a promising way to obtain resistance to both pathogens in walnut. Several co-transformed transgenic J1 and RR4 lines were recovered that showed resistance to both crown gall and nematodes. A similar inhibition by gene-silencing was observed recently in RLNs *P*. *thornei* and *P*. *zeae* grown in carrot mini discs and exposed to double-stranded RNA via soaking. Silencing of the *pat*-*10* and *unc*-*87* genes of *P*. *thornei* reduced reproduction by 77–81% [22]. Our results suggest a 1:1 concentration ratio is optimal for co-transforming walnut somatic embryos. When we employed conventional transformation using only one vector, we obtained much higher transformation efficiency. Absence of competition during transformation likely accounts for this elevated efficiency.

This research represents the first RNAi experiments involving a migratory plant parasitic nematode, *P*. *vulnus*, by artificial feeding on transgenic walnut roots. In the experiments described here, nematodes were isolated after two months of feeding on the transformed walnut roots. Several co-transformed and single-vector transformed walnut roots were used for feeding experiments. In co-transformed lines only, rolABC genes were introduced by the resident T-DNA in the *A*. *rhizogenes* Ri-plasmid used as a vector for plant transformation but not in the single-vector transformed lines with only Pv010. In this research, RNAi was used as a tool to suppress specific *P*. *vulnus* genes. The Pv010 gene sequence was chosen for these RNAi experiments based on its similarity to a *C*. *elegans* gene (prp-8) whose expression has been successfully suppressed in multiple experiments, resulting in reduced fecundity (e.g., sterility, embryo lethality, larval lethalality, etc.) relative to untreated nematodes [35-39]. Pv194 gene was chosen for its potential to act as a negative control because RNAi silencing of its *C*. *elegans* ortholog (ttr-51) has resulted in wild-type phenotype. In migratory nematodes such as *P*. *vulnus*, the major observable phenotype is population growth, which is related to the nematodes’ fecundity and ability to feed. All motile stages (J2 through adult) feed on plant cells. Although juvenile stages may feed ectoparasitically on root hairs, once the nematode has penetrated the root, feeding is restricted almost entirely to the root cortex [40]. Any paralysis or immobility will affect their feeding ability and subsequent survival or reproduction in a host. Interestingly, the *C*. *elegans* ortholog of Pv010 is a spliceosome subunit (prp-8) with RNAi phenotypes including sterility and embryo/larval lethality [35-39]. After observing similar phenotypes in *P*. *vulnus*, we can infer that this function is conserved across nematode taxa. However, the prp-8 sequence is sufficiently dissimilar to that of plant splicing factors that no detrimental phenotype was observed in the transformed walnut roots.

More work is needed to determine whether the expressed RNA has interfered with juvenile development (progression from J1 to J2 to J3, etc.) or with egg production. Independent of the transformation method, when dsRNA was present we obtained successful nematode control in some transgenic lines, suggesting that silencing the Pv010 gene orthologous to *C*. *elegans* prp-8 is very effective in controlling root lesion nematodes. When the Pv010 RNAi construct alone was transformed into walnut, at least three transgenic lines showed even higher Pv010 gene expression than line 68 and equal or better control of nematodes.

All virulent *A*. *rhizogenes* strains possess a large root-inducing (Ri) plasmid [41] which introduces rol genes when used for transformation. PCR confirmed the insertion of rol genes into co-transformed embryos lines. Our nematode assay results show that, independent of the rolABC genes inserted into co-transformed lines, we obtained up to 79% nematode reduction in co-transformed lines and up to 100% reduction in three single-vector transformed lines. Even though rolABC genes are present in the roots of co-transformed lines and changed root morphology, these rol genes do not affect nematode feeding. While there is some evidence of altered rooting characteristics in transgenic fruit trees expressing rolABC genes [42-45], there is no evidence that rol genes affect RLN control in walnuts. This is demonstrated by having a higher nematode population in line 66-1-2 which is a co-transformed J1 transgenic line possessing iaaM, ipt, Pv010 and rolABC genes. Line 66-1-2 had only 10% nematode control. Line 68-3-1 also has the same inserted genes as line 66-1-2 and had nematode control up to 79%. The single-vector transformed lines without rol genes reduced nematode population by 64-100%, depending on line. This shows that, independent of the rolABC genes, the Pv010 RNAi construct has the ability to control nematode population in walnuts. Based on the *C*. *elegans* ortholog ttr-51 RNAi phenotype (wild-type), it was predicted that Pv194 RNAi should not reduce nematode populations and there was no significant difference in nematode numbers per root between lines transformed with Pv194 (8-1-1) and untransformed controls, showing that neither the transformation procedure itself nor silencing this putative gene affected nematode survival. Our results suggest that *C*. *elegans* RNAi phenotypes may be good predictors of *P*. *vulnus* RNAi efficiency. This would simplify choosing other targets for suppression of *P*. *vulnus*.

The 400 bp Pv010 RNAi fragment inserted into walnut successfully interfered with nematode reproduction in some transgenic lines, leading to as much as 79% nematode control in co-transformed and 100% in some single-vector transformed roots with this fragment. Similar results were obtained when four different RNAi gene silencing constructs were used to transform soybean roots [46,47]. All four constructs decreased the number of mature soybean cyst nematodes at 30 days after infection by over 75%. *In planta* delivery of the RNAi fragment to the nematode provides continued exposure of the nematode to the RNAi fragments as the nematode feeds [48]. Two *Meloidogyne incognita* genes (a splicing factor and an integrase) were successfully silenced in nematodes feeding on RNAi-transformed tobacco roots [29]. The galls that formed on the RNAi-transformed roots were significantly smaller in size and number than those in untransformed control plants. Silencing of the *M*. *incognita* gene encoding cathepsin L-cysteine, mi-cpl-1, using an 800 bp fragment reduced the number of females that could produce eggs by 60% when the nematodes were soaked in an octopamine solution 21 days after infection [49]. The dsRNA may be ingested by the feeding nematodes through the feeding tubes. Alternatively, dsRNA molecules are processed by the plant RNAi machinery and siRNA are ingested [4]. Thus, it was reasonable to assume that the gene fragments we used could also be taken up by the nematode.

We observed phenotypic differences in walnut plants transformed with rolABC genes. While our constructs had no rolABC genes in the T-DNA, those three genes were inserted when we used *A*. *rhizogenes* for co-transformation. Shoot and root phenotypes were consistent with the expected effects of inserted rolABC genes [42-44]. RolA induces wrinkled, slightly curled leaves and dense, bushy foliage in transgenic walnut plants. RolB is considered the most important rol gene for root induction. RolB increases rooting potential. In many plants, introducing rolB alone efficiently induced fast-growing, highly branched, and plagiotropic roots. The thicker, well-developed root system observed in transgenic *in vitro* plantlets is due to rolB, which increases tissue auxin sensitivity and alters leaf morphology [50]. Our transformed plants also had shorter internodes in shoots and increased branching in roots due to the insertion of rolC. Similar results were obtained in walnut shoots [45]. The root phenotypic characters reported here conflict with a previous report of few, fibrous roots in walnut plants transformed with rolABC [45]. Our *in vitro* walnut plantlets had abundant healthy, long, thick roots.

We have demonstrated here that similar to other plant parasitic nematodes studied, the RLN *P*. *vulnus* is amenable to dsRNA-mediated RNAi via feeding on transgenic plant materials containing dsRNA. Therefore, RLNs, which are migratory endoparasites, can potentially be controlled using this technique. This result agrees with the recently published *P*. *thornei* and *P*. *zeae* results using double stranded RNA-induced gene silencing via soaking [22] and transcriptomes analysis of *P*. *thornei* and *P*. *coffeae* to confirm the presence of an efficient exogenous RNAi pathway and mechanism [22,51,52].

## Conclusions

Silencing the genes encoding iaaM, ipt, and Pv010 can greatly decrease crown gall formation and simultaneously reduce root lesion nematode infestations in walnuts. The most efficient co-transformation was achieved when bacterial mixtures were combined in similar concentrations. A transformation system using only one vector was more effective than co-transformation in achieving higher control of RLNs. The *C*. *elegans* ortholog of Pv010 is a spliceosome subunit whose RNAi phenotype includes sterility or juvenile lethality in *C*. *elegans*; our results suggest Pv010 suppression has a similar effect on *P*. *vulnus*. Pv194, as predicted based on knowledge of its *C*. *elegans* ortholog, did not adversely affect *P*. *vulnus* populations. These results suggest *C*. *elegans* RNAi phenotypes may be good predictors for *P*. *vulnus* RNAi results, which would help greatly in choosing other targets for *P*. *vulnus* suppression. Viable resistance to root lesion nematodes in crop plants may be achieved in the future using RNAi technology. More research is still needed to determine if the construct used in this work has interfered with juvenile development or egg production.

*P*. *vulnus* feeding experiments and crown gall testing of the transformed RR4 and additional transformed J1 embryo lines are underway. After successful characterization of those lines, they will be micropropagated as walnut shoots, rooted, and the *in vitro* results will be confirmed in greenhouse and field tests. Studying nematode population dynamics and gene expression following digestion of dsRNA will provide additional information for successful control of root lesion nematodes in walnuts.

## Methods

### Plant materials, vectors and bacterial strains

All plant material used in this research was from previously established walnut somatic embryo cultures. Somatic embryo line J1 was derived from a single open-pollinated immature zygotic embryo of *J*. *hindsii* 'James’ x *J*. *regia*. Somatic embryo line RR4 originated from an open-pollinated *J*. *hindsii* 'Rawlins’ x *J*. *regia* cross. Both lines were initiated using the procedures of [53] and have been propagated in culture by direct somatic embryogenesis for many years using standard methods [53,54].

**Figure 7:**
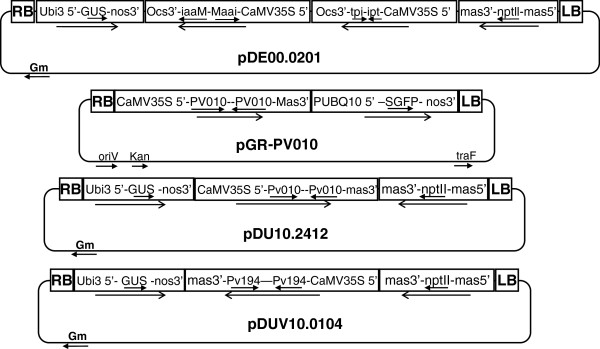
**Binary vectors used for walnut transformation.** The *Agrobacterium* binary vectors pDE00.0201, for expression of self-complementary iaaM and ipt oncogenes, and pDU10.2412 and pDUV10.0104, for Pv010 and Pv194 gene silencing, are shown. The pGR-Pv010 vector was used for co-transformation of walnuts with vector pDE00.0201. Arrows indicate the direction of transcription. LB and RB indicate the left and right T-DNA border sequences.

The *P*. *vulnus* genes chosen for RNA interference experiments and the plasmids used are described (Figure 7). All vectors were obtained from the laboratory collection of Abhaya Dandekar, University of California, Davis. The co-transformation experiments designed to yield both crown gall and *P*. *vulnus* resistance used *A*. *tumefaciens* binary vector pDE00.0201 [30,31] and *A*. *rhizogenes* vector pGR-Pv010. Plasmid pDE00.0201 carries the iaaM, ipt, GUS (β-glucuronidase), and nptII genes, while pGR-Pv010 carries Pv010 and GFP but no selectable marker gene. A second approach using single-vector transformations directed at achieving only *P*. *vulnus* RNAi employed *A*. *tumefaciens* binary vectors pDU10.2412 and pDUV10.0104 containing the Pv010 and Pv194 genes, respectively, along with GUS and nptII.

### Generation of transgenic RNAi somatic embryos

Walnut somatic embryos were co-transformed via the mixture method, using *A*. *tumefaciens* EHA101 and *A*. *rhizogenes* MSU440 harbouring binary vectors pDE00.0201 and pGR-PV010, respectively. Bacteria from cryogenic freezer stocks were streaked onto plates of 523 media [55] and incubated 24 hours at 28°C. Liquid cultures were initiated from plates by transferring bacteria from a single colony to a 50 mL tube containing 20 mL 523 liquid medium with the appropriate antibiotics added to ensure retention of plasmids. Liquid cultures were then grown overnight at 26-28°C [55], centrifuged (3,000 × g, 10 min) to pellet the bacteria, and resuspended to a density of 2.5 × 10^8^ cells/mL in DKW medium [53] containing 100 μM acetosyringone and 1mM proline (pH adjusted to 5.2). After adjusting the bacterial density, EHA101/pDE00.0201, containing crown gall RNAi genes iaaM and ipt, and MSU440/pGR-PV010, containing *P*. *vulnus* RNAi gene Pv010, were mixed in proportions of 1:1 or 1:3. These mixtures were used to inoculate ~75 small (2–5 mm) white, intact walnut somatic embryos each from lines J1 and RR4. In a second approach, EHA105/pDU10.2412 (Pv010) and EHA105/pDUV10.0104 (Pv194) binary vectors were used separately to transform J1 somatic embryos.

Embryos were soaked 15–20 min in the bacterial inoculum, blotted lightly on sterile filter paper, and plated on solid basal DKW medium containing 100 mM acetosyringone. Ten embryos were placed on each plate. Each initial (E_0_) embryo was given an identification number to track it and its progeny throughout the experiment. After 48h, embryos were transferred to solid DKW selection medium containing timentin (200 mg/L) and kanamycin (200 mg/L) and were maintained in the dark at room temperature. For the first two weeks, embryos were transferred to fresh medium every 2 to 3 days to prevent bacterial overgrowth. After two weeks, embryos were transferred to fresh selection medium every 7 to 10 days.

A previously described labelling system was used to distinguish among embryo generations [56]. The inoculated explants were designated E_0_ embryos, the initial secondary embryos were E_1_ embryos, and subsequent generations of embryos were called E_2_, E_3_, etc. E_1_ embryos were transferred into separate plates containing selection medium and allowed to grow. Actively proliferating, white, healthy, E_2_ embryos were used to evaluate GUS gene expression. Secondary embryos were harvested at the end of the same culture period in which they first became apparent and sorted into transformation events based on their spacing. Embryos found within 5 mm of one another were considered to be derived from the same transformation event, while embryos spaced further apart were presumed to represent separate transformation events [57].

### Characterization of transgenic embryos

Expression of the GUS gene was determined using an X-Gluc (5-bromo-4-chloro-3 indolyl glucuronide) histochemical assay (58, 59). X-Gluc is a substrate which produces a localized blue precipitate in cells expressing the GUS gene. X-Gluc substrate solution was prepared by dissolving X-Gluc to a 0.3% v/v solution in dimethylformamide. This was diluted to 1 mM X-Gluc with 100 mM sodium phosphate buffer (pH 7.0) containing 0.006% Triton-× 100 and 0.5 mM K^+^Fe cyanide. E_0_ and E_1_ embryos were maintained and secondary embryos (E_2_) were removed for evaluation at one- to two-weeks intervals, starting seven to eight weeks post inoculation. After five to six weeks, all dead and non-proliferating E_0_ embryos were discarded. Embryo line GUS 242 was obtained from the UC Davis Walnut Improvement Laboratory and used as a GUS-positive control.

E_2_ embryos were tested for expression of the GUS gene by cutting a small piece from each embryo and immersing it in X-Gluc solution in 96-well plates at room temperature. Embryos or pieces were observed for blue color at intervals beginning 10 min after immersion for up to 24 h. If the piece developed the distinctive blue color, the embryo from which it was cut was separated and multiplied on appropriate medium for further analysis.

GUS activity was measured quantitatively with a fluorometric GUS analysis using 4-methyl umbelliferyl glucuronide (MUG) to quantitatively measure GUS activity. Ten to 100 mg embryo tissue was homogenized in protein extraction buffer and the fluorescence of the protein extract in the presence of MUG substrate was quantified using a TKO100 fluorometer [58,59].

GUS-positive, actively proliferating embryos were randomly picked from different embryo lines and used for DNA isolation. Total DNA was isolated using a DNeasy Plant Mini Kit (Qiagen, Valencia, CA) according to the manufacturer’s protocols. PCR was performed with 2.5 μL 10X PCR buffer containing 1.5 mM MgCl_2_ (Applied Biosystems, Foster City, CA), 1.25 μL primers 1 and 2, 0.5 μL dNTPs, and 0.2 μL Taq DNA polymerase (Applied Biosystems). Each reaction mixture was 25 μL.

Primers used for detection of nptII were (5′-->3′):

Aph3: ATGATTGAACAAGATGGATTGCACGCA and

Aph4: GAAGAACTCGTCAAGAAGGCGATAGA

Primers used for detection of Pv010 were (5′-->3′):

Pv010: CTTATCTGATCGCTTCCTTGGC and

Pv010: AAACTTCCAATGGTCGAATAAATTC

Primers used for detection Pv194 were (5′-->3′):

Pv194: TACTCAACCACAAAATTGTCCACC and

Pv194: ATGATGGCATTATGCCGGGA

Amplifications were carried out in a Gene Amp PCR System 9700 (Applied Biosystems, Foster City, CA) as follows: pre-cycling for 2 min at 94°C, followed by 40 cycles of 1 min at 94°C, 1 min at 60°C, and 1 min at 68°C. PCR products were electrophoresed using 0.8% agarose gel, stained with SYBR safe DNA gel stain, and visualized with a UV illuminator. Bands showing 400 bp were considered Pv010- and Pv194-positive and bands showing 790 bp were considered nptII positive. DNA samples of co-transformed J1 and RR4 somatic embryo lines that were positive for Pv010 were tested for the presence of rolABC genes. The band sizes expected were 270, 775, and 540 bp for rolA, rolB, and rolC, respectively.

Rol gene primer sequences were (5′-->3′):

(rol a: F): AGA ATG GAA TTA GCC GGA CTA and

(rol a: R): GTA TTA ATC CCG TAG GTT TGT TT

(rol b: F): G GAT CCC AAA TTG CTA TTC CTT CC and

(rol b: R): GGC TTC TTT CTT CAG GTT TAC TGC

(rol c: F): G GCT GAA GAC GAC CTG TGT TCT CTC and

(rol c: R): A GCC GAT TGC AAA CTT GCA CTC GCC

PCR products of Pv010-inserted samples were sequenced at Davis Sequencing (Davis, CA) using the primers (Pv10: F): 5′-CTTATCTGATCGCTTCCTTGGC-3′ and (Pv010: R): 5′-AAACTTCCAATGGTCGAATAAATTC-3′. DNA regions of low quality sequence were manually removed from each read by visual inspection of the chromatogram using Sequencher version 4.9 (Gene Codes Corp., Ann Arbor, MI); the 5′ sequences were aligned to form a consensus sequence of 400 bp.

### *P*. *vulnus* resistance screening

Pv010 gene-positive, co-transformed J1 and RR4 lines, single-vector transformed J1 lines and Pv194 gene-positive J1 line 8 (8-1-1), and J1 non-transgenic embryos, all with well-developed cotyledons, were desiccated in dry microtiter plates for about one week at room temperature. The desiccated embryos were rehydrated on solid DKW basal medium and maintained in the dark for about two weeks at ambient temperature. When the embryos initiated roots, they were transferred to culture tubes containing 30 mL DKW medium. A rapid nematode resistance screening assay was used to test nematode multiplication in transformed roots [60]. Nematodes used in this research were from a population of *P*. *vulnus* originally isolated by the Howard Ferris Lab, Department of Nematology, UC Davis, from soil collected at a northern California walnut orchard and maintained *in vitro* on walnut roots in the lab of Gale McGranahan (Department of Plant Sciences, UC Davis). These nematode cultures have been maintained *in vitro* for ~20 years.

Nematodes were collected from *in vitro* cultures under sterile conditions using 20 mL plastic syringes as described [60]. In a laminar flow hood, *in vitro*-grown, two-month-old walnut roots containing lesion nematodes were cut into 1 cm pieces and three pieces were placed in each of three sterile syringe tubes. The tubes were then filled to the 5 mL line with sterile water, capped, sealed with Parafilm, and kept at room temperature in the dark for one day. The root pieces were then removed from the syringe tubes in a laminar flow hood. The number of nematodes in each tube was determined by counting three 0.25 mL sub-samples under a microscope and the volume needed to obtain 100 nematodes was calculated.

The liquid with 100 suspended nematodes was then pipetted onto each rooted embryo in a capped glass culture tube and incubated at room temperature in the dark for about two months. There were three biological replicates from each transgenic and control line. The entire experiment was repeated twice at different times.

After two months of co-cultivation, the total nematode population in each tube was collected using Baermann funnels. One folded Kimwipe (Kimberly-Clark) was placed on each funnel to act as a sieve. Each rooted embryo, along with its culture medium, was removed from the culture tube. The roots were chopped into small pieces and both roots and medium were put on top of the water-filled funnel apparatus to let the nematodes move into the water. Each culture tube was rinsed with water several times and the rinse water added to the funnel to make sure all nematodes were recovered. Enough water was added to each funnel to make sure the roots and medium were in constant contact with the water while the filter equipment was kept at room temperature in the dark for about three days. Fifteen mL liquid was collected from each filter daily and placed in a capped plastic 15 mL centrifuge tube; the funnel was refilled with water back to replacement level. The liquid collected from each funnel was centrifuged and adjusted to a volume of 5 to 10 mL. The number of nematodes present in each tube was calculated from counted subsamples. After thorough mixing, a 250-μL suspension was taken for counting and this was repeated three times to reduce counting errors. Nematode counts were analyzed using a Poisson mixed generalized linear model, in which a random sample effect was used to avoid the effects of overdispersion in the data. Posthoc comparisons against the control treatment were done using a Dunnett-Hsu adjustment, and pairwise comparisons among the non-control treatments were done using a Tukey-Cramer adjustment.

### Crown gall resistance screening

Transgenic embryo line 33-3-1, which was positive for both crown gall and Pv010 genes, and untransformed J1 control embryos were desiccated over a saturated ammonium nitrate solution [61] and germinated on basal DKW medium. Shoots emerging from germinated somatic embryos were excised and multiplied by micropropagation [62]. Sixteen microshoots of each transformed 33-3-1 line and untransformed J1 control were used as explants. The virulent *A*. *tumefaciens* strain 20W-5A was used for the tumorigenesis assay [30]. Microshoots were inoculated with bacteria at a cell density of 2.5 × 10^8^/mL. Inoculated shoots were first cultured on DKW media containing 100 μM acetosyringone for 48 hours. After two days, shoots were cultured on DKW containing 200 mg/mL timentin to inhibit bacterial overgrowth. The incidence and mass of undifferentiated callus tissue generated at *A*. *tumefaciens* inoculation sites were assayed five weeks post-inoculation. Small (2 to 3 mm) tissue slices were excised from the surface of induced callus and cultured on hormone-free DKW. Hormone-independent tissue growth was assayed after five weeks.

## Competing interests

The authors declare that they have no competing interests.

## Authors’ contributions

AMD and SLW designed the study. MTB designed and built the pGR-Pv010 and Pv194 plasmids. CAL, MTB and SLU coordinated the project. YYL and CAL developed the nematode *in vitro* co-culturing procedures. SLW performed the experiments and wrote the manuscript. All authors read and approved the final manuscript.
